# A Secure 3-Way Routing Protocols for Intermittently Connected Mobile Ad Hoc Networks

**DOI:** 10.1155/2014/865071

**Published:** 2014-07-17

**Authors:** Ramesh Sekaran, Ganesh Kumar Parasuraman

**Affiliations:** ^1^Anna University Regional Centre, Madurai, Tamil Nadu 625007, India; ^2^K.L.N. College of Engineering, Sivagangai, Tamil Nadu 630611, India

## Abstract

The mobile ad hoc network may be partially connected or it may be disconnected in nature and these forms of networks are termed intermittently connected mobile ad hoc network (ICMANET). The routing in such disconnected network is commonly an arduous task. Many routing protocols have been proposed for routing in ICMANET since decades. The routing techniques in existence for ICMANET are, namely, flooding, epidemic, probabilistic, copy case, spray and wait, and so forth. These techniques achieve an effective routing with minimum latency, higher delivery ratio, lesser overhead, and so forth. Though these techniques generate effective results, in this paper, we propose novel routing algorithms grounded on agent and cryptographic techniques, namely, location dissemination service (LoDiS) routing with agent AES, A-LoDiS with agent AES routing, and B-LoDiS with agent AES routing, ensuring optimal results with respect to various network routing parameters. The algorithm along with efficient routing ensures higher degree of security. The security level is cited testing with respect to possibility of malicious nodes into the network. This paper also aids, with the comparative results of proposed algorithms, for secure routing in ICMANET.

## 1. Introduction

The era of network started with the traditional wired networks, that is, lasting from many decades, that communicate through physical medium. It leads to wireless networks where communication does not enfold the physical medium. Another form of network evolved where nodes are intended to move within the network named mobile networks. Subsequent to which is the wireless sensor networks (WSN) where the communication channels operate through the inbuilt wireless sensor devices within the nodes. A new form of network raised as a challenge for routing called ad hoc network with a special feature of dynamically changing topology. Mobile ad hoc networks (MANET) [1–4] run through into existence where the topology keeps changing frequently in addition to the mobile nature of nodes. At present, the intermittently connected mobile ad hoc networks (ICMANET) [5] explored a new era where the connectivity between nodes never occurs with high density of nodes resulting in spasmodic environment.

The routing in all forms of networks is possible through traditional routing schemes like distance vector routing, link state routing (LSR), open shortest path first (OSPF), opportunistic adaptive routing, distance source routing (DSR) [6], ad hoc on demand distance vector (AODV) [7], and destination-sequenced distance vector (DSDV) [8]. But these schemes are not applicable for ICMANET.

In general ICMANET is a delay tolerant network (DTN) [9, 10] capable of holding larger delays. It is designed to operate effectively over extreme distances such as those encountered in space communications or an interplanetary scale. The sparse or dense nature of intermittent network is mainly due to high mobility of the nodes. The nodes stir within fractions of time and hence their topology changes in a dynamic way. Typical examples of intermittent network are wild life tracking, habitat monitoring sensor networks, military networks, nomadic community networks, vehicular networks, and so forth. Due to typical distorted nature of the network, routing becomes an onerous task.

Many routing algorithms are proposed for efficient routing in the intermittent network, namely, flooding [5], epidemic [11], direction based routing, adaptive routing, utility based routing, probabilistic routing, copy case routing, spray and wait [12] routing, and so forth. These algorithms furnish a proficient channel for data transmission. But they do not clear out a way for efficient secure routing.

The secure communication in MANET adheres to enormous technical challenges due to unique characteristics of MANET. In addition to that it opens for eaves dropping due to intermediate communication and also holds many security threats. Hence security in MANET is adopted using intrusion detection system (IDS) or by creating a trust [13] based environment. The IDS shows extended varieties like behavior based IDS [14], knowledge based IDS [14], distributed IDS [15], real-time IDS [15], multilayer integrated anomaly detection system [4], clustering approach [16], mobile based detection system [17], cooperative approach, [18] and so forth. These known systems enhance the secure communication in MANET.

The security protocols designed for MANET are not suited for ICMANET which is mainly due to the disconnected nature of the network. In the trust based system all IDS must comprise a central server to ensure a node's authentication. The impossibility of setting central server in ICMANET does not adopt these techniques. Hence a new mode of security protocol is to be designed that does not demand for a central system.

The target of secure routing is to prevent malicious attacks by the intruders. This also prevents unwanted attacks or threats of data in the network. The secure routing should be provided in an efficient manner such that they should not degrade the normal performance of data routing in the networks, that is, increase in delay, higher overhead, and maximum storage capacity; invariant bandwidth should not occur. In this paper, we put forth optimum secure algorithms, namely, LoDiS routing with agent AES (LA), A-LoDiS with agent AES (A-LA) routing, and B-LoDiS with agent AES (B-LA) routing that aims at providing a high range of security with optimum result than the existing protocols. The normal routing with ant and bee operates at milliseconds whereas on agent setup the routing operates at nanoseconds and hence the delay in performing the authentication process will be maintained at an ordinal form. This ensures no degradation in the performance of routing. In this way, an efficient secure communication is proposed here. This paper also frames that B-LA provides the better result in contrast to LA and A-LA. The degree of security is measured with introducing malicious nodes into network and ordeal with the three proposed algorithms.

The data transmission in each proposed scheme undergoes diverse algorithms. LA, A-LA, and B-LA employ location aware routing for delay tolerant networks (LAROD) routing, ant colony optimization (ACO) technique, and bee colony optimization (BCO) technique, respectively. All the three algorithms ensure security by means of agent setup and cryptographic technique. All nodes in this network have agent set within them. The agent performs three tasks in prior to data transmission, namely, node analyzing, data aggregation, and data broadcasting. The data aggregator is a simple database that holds all the data regarding the respective node. Each node has unique id, passcode, origin, grid card representation, and specific pattern. All these information along with the mobility model are stored in the data aggregator of each node. The node analyzer tests whether a node is malicious node based on these agent parameters mentioned above. Once a node is assured for a trusted node the data broadcaster broadcasts the data packet to the nearest possible node. The agent setup imposes secure communication, to propose secure data transmission; the advanced encryption standard (AES) algorithm is used. This algorithm is mainly chosen for its efficiency in handling timing and power attacks. In depth comparison is made between these three algorithms to show the best of the optimum results delivered by them.

The paper is prearranged as the following sections.  Section 2 describes the work related to ICMANET.  Section 3 portrays the mechanism of secure routing in LA, A-LA, and B-LA. The simulation results are depicted in the Section 4.

## 2. Related Work

The Intermittently connected network is a new form of emerging network where routing data packets is seemed to be monotonous task. Many research works have proved the possibility of routing in ICMANET. This section provides an overview of routing techniques applicable in the intermittent network. It also conveys the general concept of ACO and BCO algorithms.

### 2.1. Routing in ICMANET

The traditional routing scheme that forms a basis for the routing schemes in ICMANET is the flooding based routing. In this, one node sends packet to all other nodes in the network. Each node acts as both a transmitter and a receiver. Each node tries to forward every message to every one of its neighbors [19]. The result in every message eventually is delivered to all reachable parts of the network.

The Epidemic routing oeuvres on the basis of the traditional flooding based routing protocol, which states that periodic pair-wise connectivity is necessitate for message delivery [2]. The protocol banks on immediate dissemination of messages across the network. Routing occurs based on the node mobility of carriers that are within distinctive position of the network.

The beaconless routing protocol [8] is grounded on the hypothesis where there never exists an intervallic diffusion of beacons into the network. Routing primarily makes a choice of forwarding node in a dispersed modus amidst its neighbors, without any form of erudition about their location or prevalence.

The context aware routing (CAR) [7] algorithm paves the forethought of asynchronous communication in ICMANET. The algorithm endows a basement of organizing the messages in the network. It addresses that the nodes are able to exploit the context information to make local decisions which imparts the good delivery ratios and latencies with less overhead. CAR is pain staked as a general framework to predict and evaluate context information for superior delivery of messages.

The Brownian gossip [6] is an amalgamation of gossip and the random node mobility which provides a scalable geographical routing. In this routing, each node forwards the query related to other nodes information with certain values of probability. Gossiping is a resourceful approach for information dissemination and is done with a probability, namely, P gossip. The probability value makes certain that the query can reach the secondary nodes in the network with highest probability.

The mobility profile based routing [1] addresses, a hub-level routing method and two versions of user-level routing methods [3]. The routing involves a SOLAR-HUB (sociological orbit aware location approximation and routing) which manipulates the user profiles that aids in hub-level routing.

The direction based geographic routing (DIG) [20] algorithm is grounded on geographic location of packets that are routed in an average approximate ideal path towards destination. The algorithm postulates that when two nodes encounter each other, the nodes exchange the knowledge of their current location, moving direction, and packets. The packets are forwarded to nodes whose distance and moving direction are closest to destination.

The single copy case routing [21]: from its nomenclature it postulates that only a single copy of message packet is carried to destination. The routing scheme includes direct transmission, randomized routing, utility based routing, seek and focus, and oracle based routing.

The multiple copy case [22] scheme deals with the mechanism of spraying a few copies of message and then routing each copy in isolated manner to the destination. The algorithm that holds multiple copy case routing are spray and wait and spray and focus.

The semi-probabilistic routing (SPR) [12] algorithm considers that the network is partitioned into tiny portions that have a stable topology. The protocol upholds the information about host mobility and connectivity changes for more accurate message forwarding.

The contention based routing postulates that the efficiency of routing can be achieved only by taking into account the contention and dead end [23]. The spray select and focus provides a better performance considering the contention and dead ends.

The spray and hop [24] is a routing protocol that holds two phases, namely, spray phase that sprays few copies of message into the network. Hop phase which occurs after the spraying phase, a node that was not able to find the destination, switches to the hop phase.

The spray and wait [11] is a scheme that sprays into the network a fewer number of message copies and waits until one of these nodes that holds the copies reaches the destination. It is simple to implement and can be optimized to achieve the depicted performance.

The LAROD-LoDiS [5] routing is a geographical routing that uses a beaconless routing protocol and a store forward carry technique. It also uses a database to communicate among them to achieve routing. It is done by Gossiping protocol. It provides constant overhead and higher delivery ratio.

### 2.2. Optimization Techniques

The algorithms adopted in this paper use certain optimization techniques. To enhance the performance of data transfer, optimization techniques, namely, ACO and BCO, are used.

ACO is a form of swarm intelligence, a relative approach to problem solving. The swarm intelligence takes a token of inspiration from the social behaviour of insects and of the other animals. In concord to this ACO [16] takes inspiration from the foraging behaviour of ant species. The general behaviour of ant involves depositing pheromone, a chemical substance used by ant to find a path in search of its food. The pheromone deposition acts as an indicator of the way to other members of the colony.

The ants in general use a stigmergy mode of communication. This communication holds two main attributes, namely, (i) an indirect nonsymbolic form of communication where insects exchange information by modifying their environment and (ii) local information which is assessed by those insects that visits the immediate neighborhood.

The general ACO technique involves initialization, traversing, pheromone deposition, and updating the pheromone.

BCO [25] is an optimization technique under the swarm intelligence, a part of artificial intelligence which is based on the actions of individuals in various decentralized systems. The decentralized system is composed of individual systems that are capable to communicate, cooperate, collaborate, and exchange information among them. BCO [26] is a “bottom-up” [27] approach. Artificial bees are created in BCO that acts as artificial agents inspired by the general behavior of natural bees aiding in the solution for optimization problems.

BCO [28] is inspired by the natural behavior of bees and is a population-based algorithm. The basic idea behind BCO is to create a group of artificial bees. The artificial bees represent agents, each generating a new solution. The process is to generate an optimal solution. The BCO algorithm consists of two phases namely the forward and backward phases respectively. The forward phase is a search phase during which artificial bees undergoes a predefined number of moves, constructing the solution and hence yielding a new solution. The new solution obtained is the partial solution. The artificial bees then start the backward phase, where they share information about their solution with each other. The information sharing is estimated by the objective value function.

The security in network plays an imperative role in preventing threats or data theft by the intruders. The secure measures are led by series of checkpoints to ensure safe data transfer. The privation for security is essential in network communication.

### 2.3. Agent Technology

The agent is a program module that functions incessantly in a meticulous environ [29]. It is proficient in carrying out activities in a supple and intellectual comportment, that is responsive to changes in the surrounding environ. The agent is not a complete program but is an interface [30] responsible for performing the preassigned chores. Agent is autonomous which takes actions grounded on its innate knowledge and its precedent experiences [31].

On setting agent at each node, security [32] is achieved by incorporating certain agent parameters at each node. The agent parameters [33] are described as follows.

- *Node ID*: a unique identifier for each node in the network.
- *Passcode*: a common password for the nodes in the network.
- *Mobility model*: the mobility model of the network topology.
- *Origin of placement*: the initial placement of each node in the network.
- *Grid card*: an *n* × *n* matrix in which each grid contains a particular data in it. Each node contains a unique grid.
- *Pattern formation*: the network is associated with certain geometric silhouettes and using these, each node encompasses a unique pattern.

These agent parameters settle on the security issue coupled in the ICMANET.

Agent is set in each node and it includes three components [33, 34], namely:

- Data Aggregator.
- Node Analyzer.
- Data Broadcaster.

*Data Aggregator.* The data aggregator is similar to a database that holds an aggregate of information about all the nodes within the network. It includes detailed information of each and every node. The aggregator holds the agent parameters of every node. In plain, it is just the collector of information.

*Node Analyzer.* The node analyzer analyses whether the node that accept the information is a family node, that is, node that belong to the topology. The analysis is based on the agent parameters that are hoarded in the data aggregator. It selects any one of the parameter in a random manner to conclude that the node is a family node. If a malicious node is sensed, the node analyzer broadcasts the presence of intruder.

*Data Broadcaster.* In the data broadcaster, once after a node is determined to be a family node, it allows the sender or any relay node to transfer the message packet to the secondary relay node. It acts as a gateway that provides access for communication amidst the encountered nodes.

**Figure 1:**
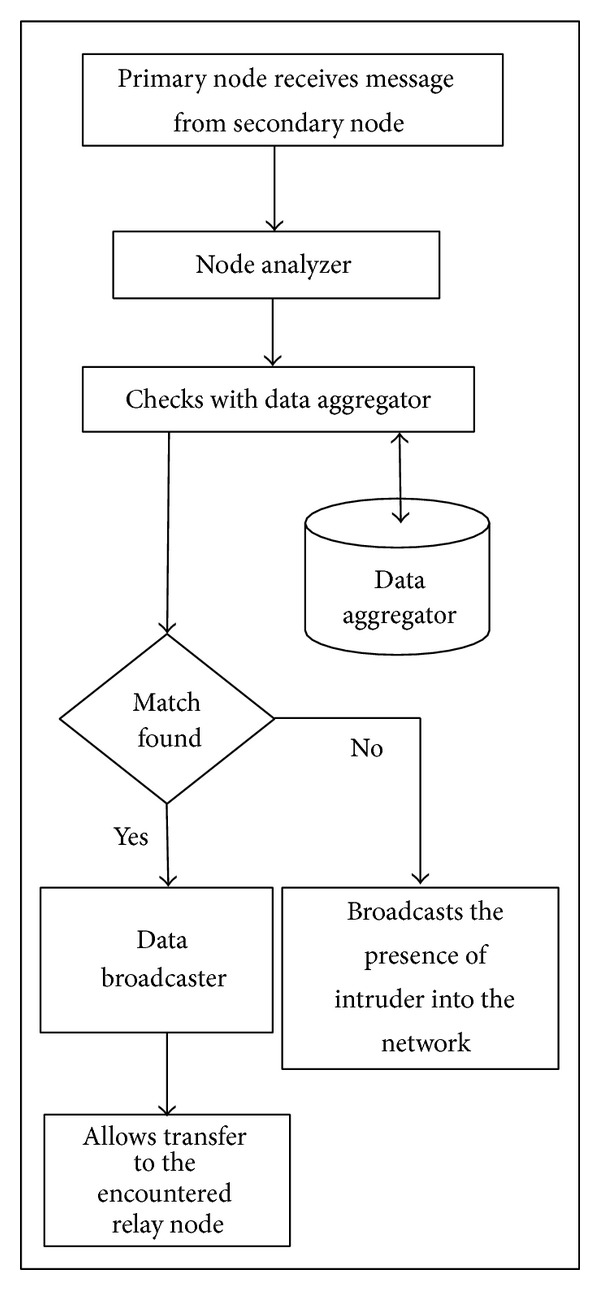
Working of agent.

From Figure 1 the architecture and working of agent set at each node. When a secondary node receives a message packet from a primary node, the node analyzer tests whether the node belongs to the home network or not. The node analyzer selects one of the parameters randomly and checks for the authorized node from the data aggregator.

The data aggregator is a database, if a match is found within the data aggregator; it is preceded towards the data broadcaster. If the node is not valid, node analyzer broadcasts the presence of the intruder within the network. The data broadcaster allows the node to transfer the message packet to the encountered node.

### 2.4. AES Algorithm

The AES algorithm is chosen mainly due to its reliable characteristics of security, cost and code compactness and its design and implementation simplicity. As AES [35] accepts data block sizes of 128, 192, and 256 and a key size of 128 bits, which can be variably expanded, it can accommodate a wide spectrum of security strengths for various application needs. Multiple encryptions use a plural number of keys, since it has been avoided in AES, a reduction on number of cryptographic keys for an application to manage is reduced and hence the design of security protocols and systems are simplified. The AES algorithm is chosen mainly for the following reasons.

- Effectiveness in high speed applications.
- Simplicity—code compactness.
- Flexible—varies with the size of input key.
- Prevent timing and power attacks.
- Used in restricted space environments.
- Cost effective.

## 3. Routing Mechanisms

In this section a secure communication with the aid of LAROD-LoDiS, A-LoDiS, and B-LoDiS routing is described. An agent is set at each node. During routing, when a sender node A wishes to transmit a data to a destination node X, it initially sends it within its boundary. Node A sends the data only if it confirms that node R1 is a trusted node within the network, that is, it is a node belonging to that particular network. The confirmation on trusted node is done using the agent technology. The agent present at each node generates a test towards R1. The node analyzer of agent at A selects one of the test parameters of agent and passes it to R1. If R1 replies with the correct reply, it is assured to be the trusted node and A passes data towards it. The data actually resides in an encrypted form and sent to R1. R1 just delivers it to another node either relay node *Rx*  (*x* = 2,3 … *n*) or destination node X. The encryption and decryption is done by AES algorithm.

### 3.1. LAROD Routing with Agent AES

In general the selections of the relay nodes are done with the help of LoDiS that uses the gossiping technique by which each node can determine the location about its immediate nodes. Hence the destination can be reached by LAROD that uses the store-carry-forward [5] and beaconless technique [5] in aid with LoDiS. Thus, a secure communication is enchanted. The pseudocode for secured LAROD-LoDiS is depicted in Algorithm 1.

### 3.2. A-Lodis with Agent AES

The selections of the relay nodes in A-LA are done with the help of Ant routing scheme using pheromones. Each node in the network acts as an artificial ant. Initially the ants (here ant refers to the node in the network) will be in the sleep mode and the pheromone value (PH) is set to 0. As a data packet is generated at a node A, it searches the relay node in a random manner. The efficiency of the relay node, that is, capability of the relay node to deliver data packet towards destination, is determined using the gossiping method. With the successful delivery of data packet by the relay node, the pH value is incremented each time. For every half minute, when a relay node is inactive, the PH value is decremented. Hence based on PH value the relay nodes are selected and are used for transmission.

LoDiS of A-LA aids in routing by means of the gossiping technique by which each node can determine the location about its immediate nodes. Hence the destination can be reached by Ant routing in aid with LoDiS technique. The pseudocode for secured A-LA is depicted in Algorithm 2 and the Ant routing for A-LA is shown in Algorithm 3.

### 3.3. B-LodiS with Agent AES

The selections of the relay nodes in B-LA are done with the help of bee routing scheme using objective value (OV). Each node in the network acts as an artificial bee. Initially the bees (here ant refers to the node in the network) will be in the sleep mode and the OV is set to 0. As a data packet is generated at a node A, it searches the relay node in a random manner. The search of relay node is a step of forward phase. The efficiency of the relay node that is capability of the relay node to deliver data packet towards destination is determined using the OV estimated during the backward phase and the gossiping method. The gossiping is mainly used to have knowledge about the positions of node in the network to transfer data through it. With the successful delivery of data packet by the relay node, the OV value is incremented each time and the efficiency of the path is shared during the backward phase.

LoDiS of B-LA aids in routing by means of the gossiping technique by which each node can determine the location about its immediate nodes. Hence the destination can be reached by Bee routing in aid with LoDiS technique. The pseudocode for secured B-LA is depicted in Algorithm 4 and the B-LA is shown in Algorithm 5.

## 4. Simulation Results

This section describes the simulation results of the proposed algorithms LA, A-LA and B-LA. The performance of these algorithms is described and they are compared with each other to highlight the best of three algorithms. B-LA exerts optimum performance. It outstands LA and A-LA. This variation is depicted in this section evidently. The comparison is made with respect to various network parameters and also it is made in contrast to the influence of the malicious nodes.  Section 4.1 clearly shows the scenario setup for evaluation.  Section 4.2 expresses the various network parameters under which the evaluation is made. The influence of number of nodes with varying parameters is portrayed in Section 4.3. The Section 4.4 insists the performance with respect to the varying transmission range. The Section 4.5 shows the influence of malicious nodes and the behaviour entrusted by LA, A-LA and B-LA.

### 4.1. Scenario Setup

The parameters set are the basic one simulator [36–38] environ parameters and are given in Table 1. The One Simulation [39], in this paper uses the random waypoint mobility model. The nodes move in an area of 2000 × 2000 m with a speed limit within bounds 0.5 to 1.5 m/s. The radio range is set to 250 m. The efficiency of any routing protocol is determined by the node density that is the total number of nodes within the set network.

The packets are generally generated with the initial setup of the simulation and holds through the overall simulation time. The time to live (TTL) or the packet life time is set as 600 s initially that are varied lately on consideration to the performance criterion. When evaluating, the simulation is run for 3000 s.

### 4.2. Parametric Measures

This section provides an insight of the various network parameters that are used in the evaluation of LA, A-LA, and B-LA.

Three main parameters are used for evaluation, namely, overhead, delivery latency, and delivery probability. To show the effect of maximum secured routing among LA, A-LA, and B-LA, it is measured with number of malicious nodes isolated from the network as well as number of packets routed through malicious nodes. These two are evaluated with respect to mobility and number of nodes in the network.

Overhead is one of the main constraints that are to be contemplated for efficient routing. Overhead is any combination of excess or indirect computation time, memory, bandwidth, or other resources that are required to attain a particular goal.

In general the latency is defined as the amount of time it takes for a packet to travel from source to destination. The delivery latency is the time interval taken for the source or any relay node to reach the destination. In general due to the sparse nature of the ICMANET, that is, due to its highly mobile nature, the time period to deliver the data packets is desirably high.

Third metric is the probability to deliver the data to the desired location at specified speed. Under these parametric considerations the protocol performance is evaluated.

### 4.3. Influence on Number of Nodes

The variations in number of nodes indict a great impact on the performance. The node density is varied from the initial setup of 50 nodes to 250 nodes. As this gets increased, each routing protocol shows the influence made by the number of nodes on overhead, delivery latency, and delivery probability. The result variations are depicted pictorially in the following graphs.

**Figure 2:**
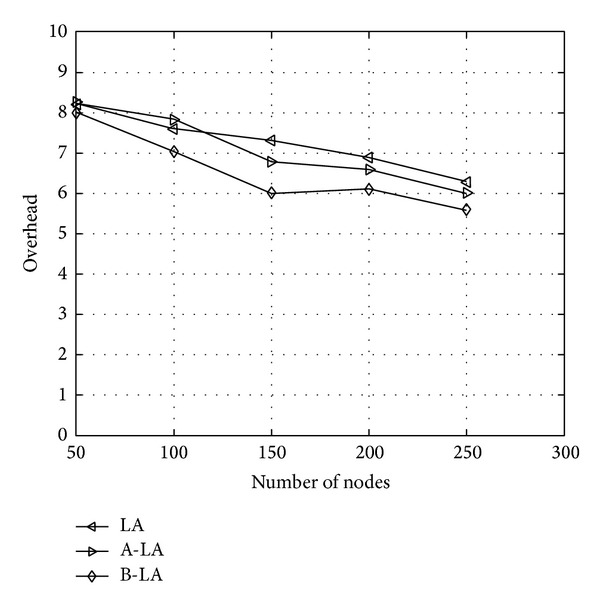
Overhead with respect to number of nodes.

The variation on overhead ratio of LA, A-LA, and B-LA are portrayed in Figure 2. The Figure 2 clearly shows that B-LA incurs a minimum overhead that is acceptable. LA and A-LA exerts slightly higher ratio of overhead than B-LA. Even though three algorithms provide optimum that is acceptable range of overhead B-LA seems to be minimum. The main reason is that B-LA uses the objective value to estimate the efficiency of the route selected. Also since it uses the backward propagation to evaluate the route it delivers minimum overhead than the other two. Figure 2 says B-LA shows 44.62% (approx) of overhead whereas LA and A-LA show a slight higher value of 57.036% (approx) and 52.87% (approx).

**Figure 3:**
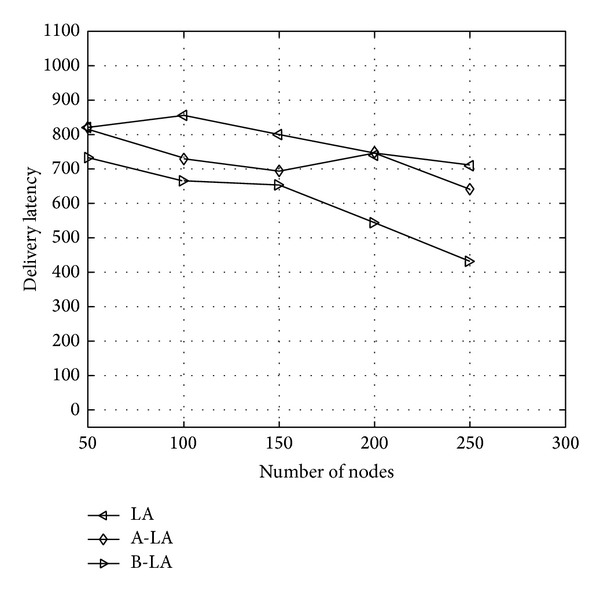
Delivery latency with respect to number of nodes.

In general the delay in ICMANET is large and the routing protocol should tolerate the delays. The LA, A-LA have trivial higher delivery delay when compared to B-LA. The Algorithm 4 clearly portrays the delay in LA and A-LA is higher when compared to that of B-LA. It concludes that B-LA exerts minimum delay in delivering data packets. From Figure 3 it is understood that B-LA exerts 34% (approx) of delay in delivering data packets. LA and A-LA incur delivery delay at a rate of 45% (approx) and 48.05% (approx). Hence B-LA results in better performance than LA and A-LA.

**Figure 4:**
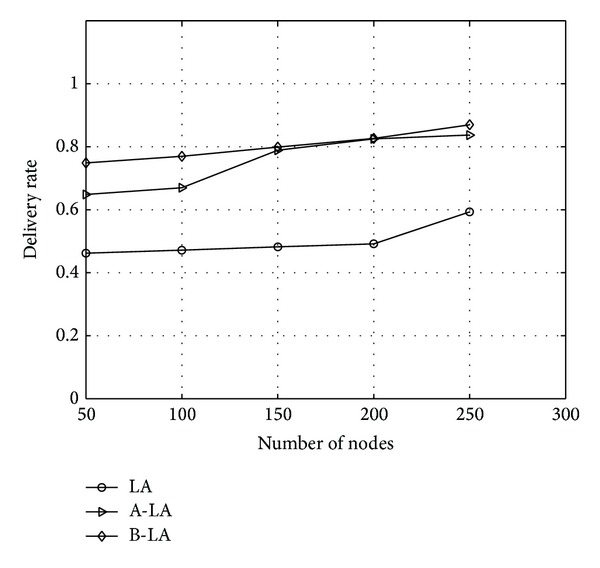
Delivery rate with respect to number of nodes.

The delivery rate in B-LA is higher when compared to LA and A-LA. The Figure 4 shows the variations on the three protocols and it evidently depicts that B-LA incurs 96.8% (approx) delivery of data packets towards the destined region. In contrast A-LA and LA deliver data packets at an average rate of 95% (approx) and 90% (approx), respectively. As a whole B-LA delivers data at better rate compared to LA and A-LA.

### 4.4. Influence on Transmission Range

**Figure 5:**
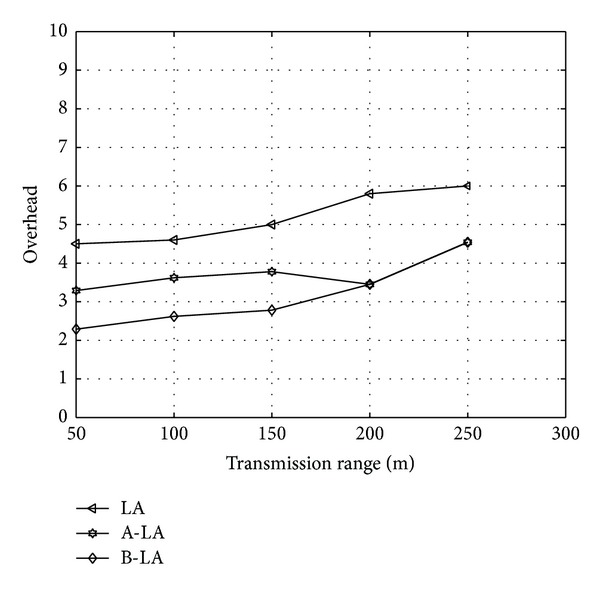
Overhead with respect to transmission range.

Transmission range has a greater impact in the behaviour of routing protocols. The three protocols LA, A-LA and B-LA are evaluated varying the transmission range from 50 to 250. The transmission range illustrates the coverage region of particular node to transmit the data packet. The metrics latency, delivery ratio and overhead are measured and compared for evaluating the higher performance among three protocols LA, A-LA, and B-LA. On varying the transmission range, the protocols show the behaviour as depicted in Figure 5. Among the three proposed protocols, B-LA wield minimum overhead compared to LA and A-LA. They vary periodically in the ratios described in subsequent. LA and A-LA have an overhead of 36.9% (approx) and 34.005% (approx). B-LA has overhead ratio at an average rate of 29.66% (approx). Hence B-LA shows minimum overhead when compared with LA and A-LA.

**Figure 6:**
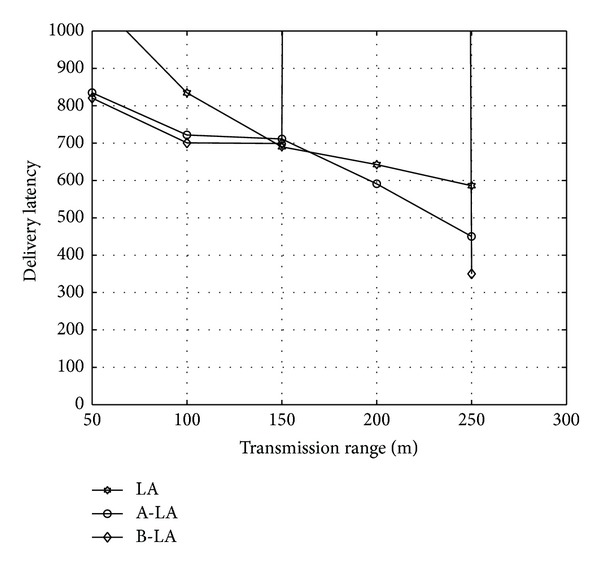
Delivery latency with respect to transmission range.

In general the delay in ICMANET is large and the routing protocol should tolerate the delays. The LA and A-LA have higher delivery delay when compared to B-LA. Since in B-LA the backward propagation ensures estimating the capacity of the route chosen to deliver the data accurately to the destined node in timely manner, B-LA has better results in contrast to LA and A-LA. The Figure 6 clearly portrays the delay in LA and A-LA is higher when compared to that of B-LA. LA has a delay of 41.48% (approx), A-LA has 37.15% (approx) whereas B-LA has 31.808% (approx) of delay in an average rate. It concludes that B-LA exerts minimum delay in delivering data packets.

**Figure 7:**
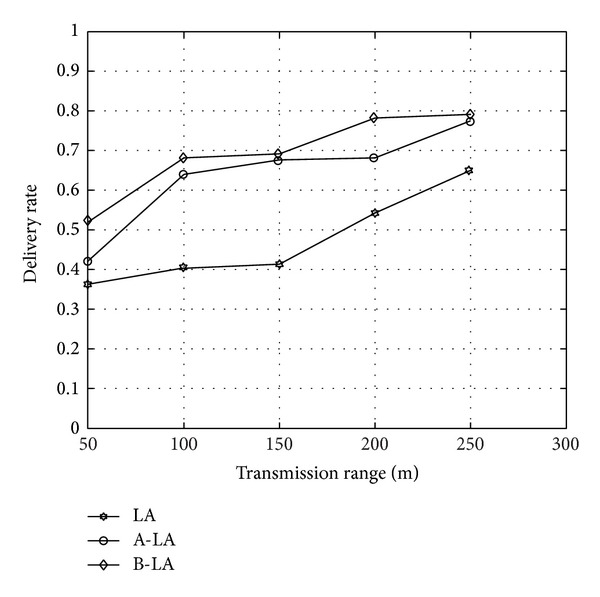
Delivery rate with respect to transmission range.

The delivery rate in B-LA is higher when compared to LA and A-LA. The Figure 7 shows the variations on the three protocols and it evidently depicts that B-LA incurs 97.09% (approx) delivery of data packets towards the destined region. In contrast A-LA and LA deliver data packets at an average rate of 94.814% (approx) and 93.73% (approx), respectively. As a whole B-LA delivers data at better rate compared to LA and A-LA.

### 4.5. Influence of Malicious Nodes

**Figure 8:**
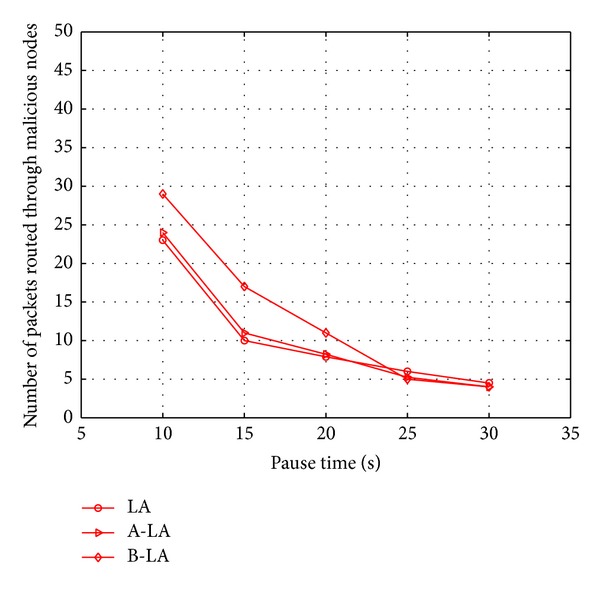
Number of malicious nodes isolated with respect to mobility.

**Figure 9:**
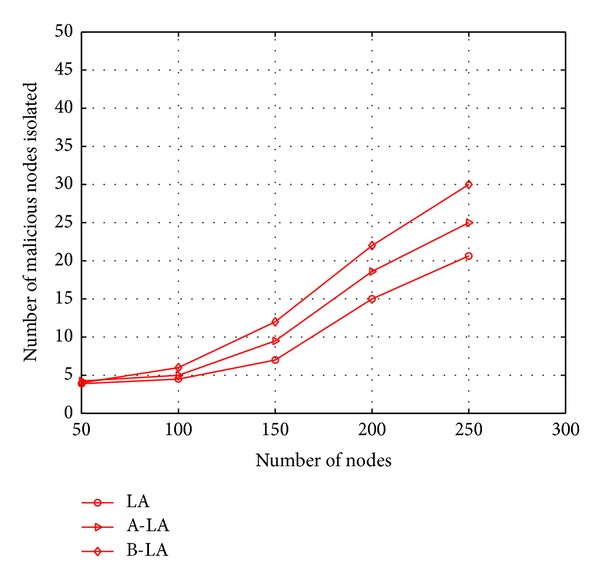
Number of malicious nodes isolated with respect to number of nodes.

**Figure 10:**
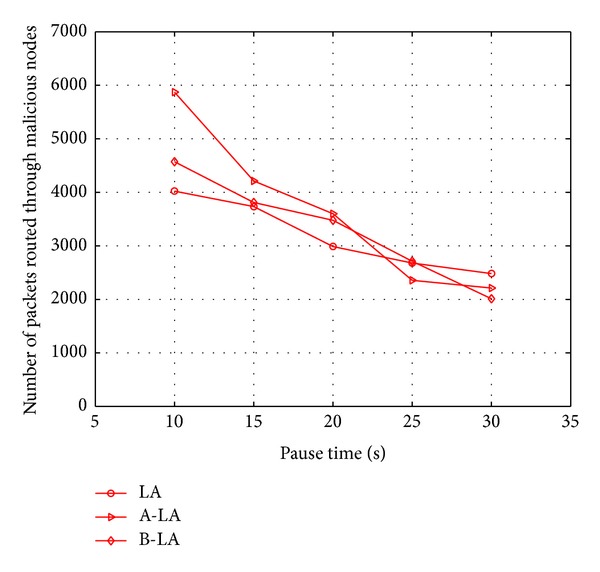
Number of packets routed through malicious nodes with respect to mobility.

**Figure 11:**
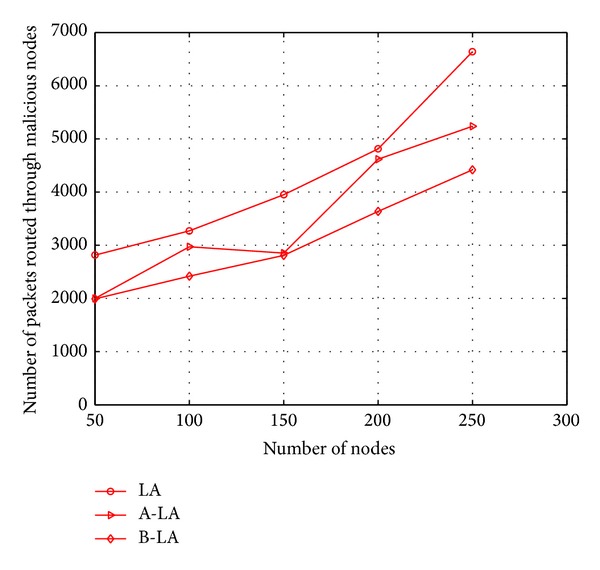
Number of packets routed through malicious nodes with respect to number of nodes.

The secure routing through LA, A-LA, and B-LA is evaluated with number of malicious nodes isolated from network and the number of packets routed through such malicious nodes. The isolated malicious nodes form network depicts the potency of secure routing scheme with detection of intruders in to the network. The Figures 8 and 9 portray the possibility of the proposed protocols to detect the malicious node in the network at higher rate. It is mainly due to the fact that these protocols use the authentication terminologies. When a node fails to meet the authentication terminologies, it is suspect to be a malicious node in the network. Here comparison is made to evaluate the best among LA, A-LA, and B-LA in secure transmission. In these protocols as the authentication scheme uses sharing process, when the number of nodes is increased in the network, more malicious nodes are detected. Figures 10 and 11 say the ratio of the protocols in detecting the presence of malicious nodes in the network with respect to mobility and number of nodes. B-LA detects at a rate of 78% (approx), A-LA detects at a rate of 76% (approx) whereas LA detects at 66% (approx) with respect to mobility. B-LA detects at a rate of 75% (approx), A-LA detects at a rate of 72% (approx) whereas LA detects at 69.66% (approx) with respect to number of nodes. This result proves that B-LA detects the malicious nodes in the network in an efficient way compared to LA and A-LA.

Considering the fact of number of packets routed through the malicious node, the probability of routing across malicious node is found to be less in B-LA compared to LA and A-LA. It is clearly depicted in Figures 10 and 11, respectively. It clearly says the ratio of the protocols in routing packets through malicious node with respect to mobility and number of nodes. B-LA detects at a rate of 38.716% (approx), A-LA detects at a rate of 33.22% (approx) whereas LA detects at 28.55% (approx) with respect to mobility. B-LA detects at a rate of 36.47% (approx), A-LA detects at a rate of 30.56% (approx) whereas LA detects at 28.88% (approx) with respect to number of nodes. This result proves that B-LA routes lesser packets across network through the malicious nodes compared to LA and A-LA. The lesser transmission of packets through malicious nodes is mainly due to the efficient detection of malicious nodes by these proposed algorithms.

Thus, these metrics clearly show that B-LA is an efficient protocol for secure routing in ICMANET among the proposed three algorithms, namely, LA, A-LA, and B-LA.

## 5. Conclusion

In this paper, we have demonstrated the efficient secure routing by means of the 3-way routing protocols LA, A-LA, and B-LA in ICMANET. The proposed routing protocols provide a higher degree of security in the intermittently connected mobile networks. The routing performance metrics are not degraded with the implementation of the security mechanism. This paper also provides a comparative analysis of the performance provided by the three algorithms and clearly predicts that B-LA paves a better way for secure transmission in ICMANET. B-LA outstands LA and A-LA with better delivery ratio, minimum overhead, and latency. It also has higher probability in detecting the presence of malicious nodes and transferring minimum data through these malicious nodes. B-LA has average overhead of 37.14% (approx) in contrast to LA and A-LA with respect to number of nodes and transmission range. It has a maximum delivery ratio of 96.945% (approx) with respect to number of nodes and transmission range. Considering number of nodes and transmission range, B-LA has a minimum delay of 32.904% (approx). It also detects proposition of malicious nodes in network and routing through it at an average rate of 76.5% (approx) and 37.593% (approx), respectively. These theoretical and analytical results prove the efficiency of LA, A-LA, and B-LA aiding in secure transmission and also pictures that B-LA provides better means of secure data transfer for ICMANET. This paper proves the efficiency of secure routing in ICMANET. As an extension to the work, multiagent will be set at each node aiding at faster and secure data transfer between nodes than setting a single agent.

## Figures and Tables

**Algorithm 1:**
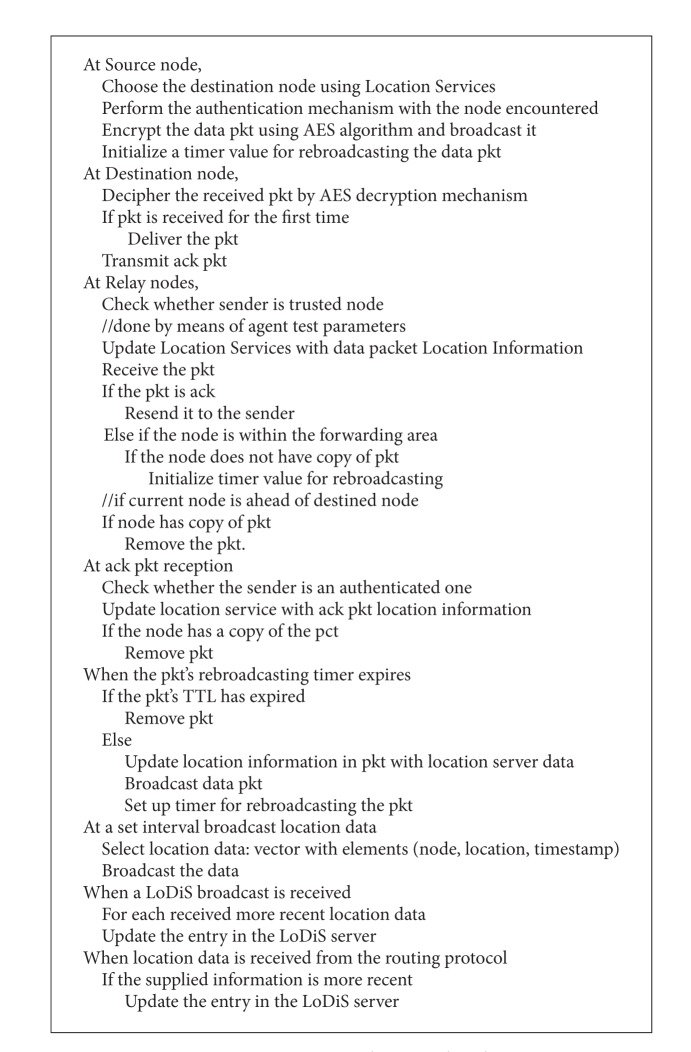
Secured LA pseudocode.

**Algorithm 2:**
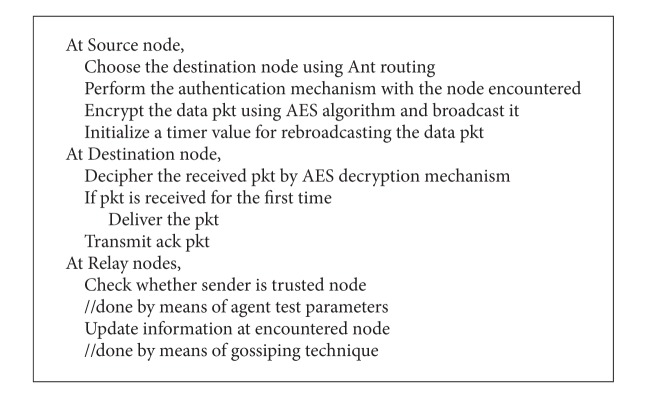
Pseudocode of A-LA routing.

**Algorithm 3:**
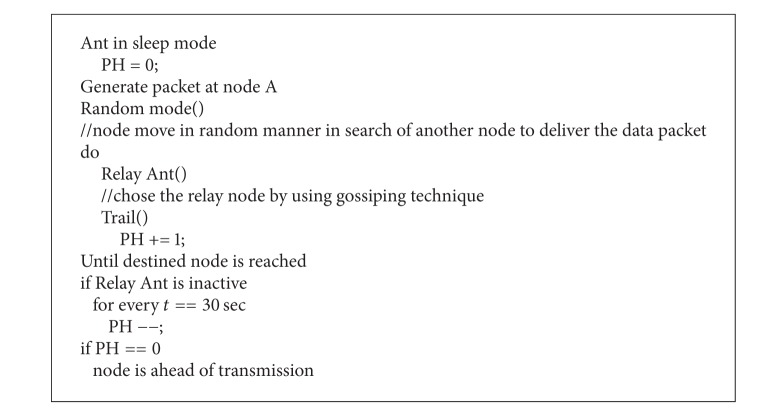
Pseudocode for Ant routing in A-LA.

**Algorithm 4:**
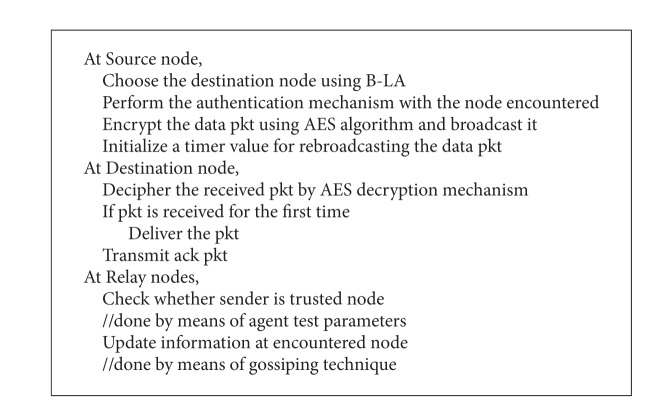
Pseudocode of secure B-LA.

**Algorithm 5:**
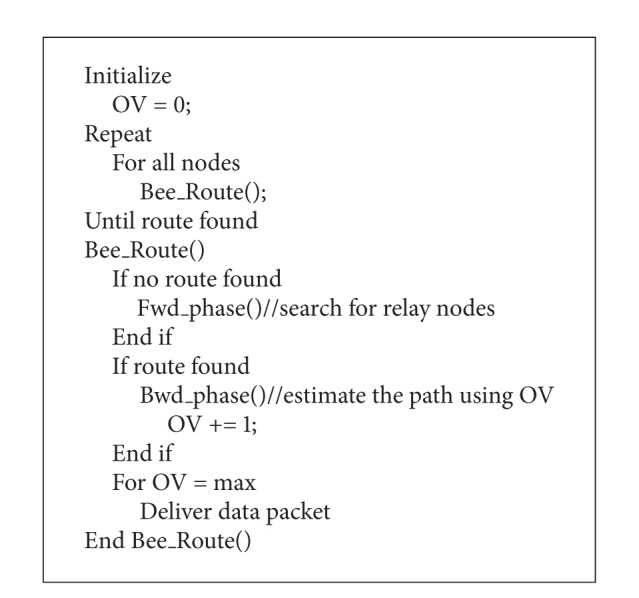
Pseudocode for B-LA.

**Table 1:** Basic simulation parameters.

Parameters	One simulator
Area	2000 × 2000 m
Mobility model	Random Waypoint
Node density	50 nodes
Node speed	1.5 m/s
Radio range	250 m
Packet life time	600 s
